# PyPeCT2S: Pythonic paediatric computed tomography to strength with automatic landmarking for the automation of bone strength analysis in children

**DOI:** 10.1371/journal.pone.0352689

**Published:** 2026-07-16

**Authors:** George Allison, Salman Almutairi, Amaka C. Offiah, Xinshan Li

**Affiliations:** 1 School of Mechanical, Aerospace & Civil Engineering, University of Sheffield, Sheffield, United Kingdom; 2 Insigneo Institute, University of Sheffield, Sheffield, United Kingdom; 3 School of Medicine and Population Health, University of Sheffield, Sheffield, United Kingdom; 4 Radiological Science and Medical Imaging Department, College of Applied Medical Sciences, Prince Sattam Bin Abdulaziz University, Al-Kharj, Saudi Arabia; 5 Department of Radiology, Sheffield Children’s NHS Foundation Trust, Sheffield, United Kingdom; Politecnico di Torino, ITALY

## Abstract

Quantitative computed tomography (QCT) based finite element analysis (FEA) models have been used to accurately predict bone strength. However, the process is time-consuming and requires a trained professional to provide manual input during several steps. The aim of this work is to automate the FEA processes of the computed tomography to strength (CT2S) pipeline applied to the paediatric femur, so that use of the pipeline requires less training, is more user-friendly and can be run for a large cohort. A deployable application was built using *Python* and *Qt* to create a repeatable, extensible, automatic, and contained platform called pythonic paediatric computed tomography to strength (PyPeCT2S), with specific attention to the development of automatic landmarking for the paediatric cohort. In this study, the computed tomography (CT) scans of 69 children were included, and FEA models were created using the PyPeCT2S pipeline. The models were subjected to four-point bending for both landmarking methods (automatic versus manual). The FEA critical moment against age results showed comparable values to existing experimental research that utilises equivalent boundary conditions, with values from 0.19–167.94 Nm. The automatic landmarking methodology was shown to produce minimal differences in location and FEA results, compared to manual landmarking, but was substantially faster to operate. The overall pipeline performance showed a mean time reduction of 49–61% and a maximum of 70% against the native pipeline, reducing completion time from 35 to 13 min. Time savings came from both process optimisations and improved user interaction pathways. The work demonstrates that a pythonic approach is a step change to speed up the prediction of FE-based bone strength while still allowing interaction and substantially limiting the chance of human error. Overall, the pythonic approach provides simpler operation and time efficiencies, allowing the tool to be used by clinicians and deployed in the clinical setting in future.

## Introduction

Child physical abuse is a large problem with upwards of 100 000 offences being recorded in the UK in 2019 [1]. Children are uniquely at risk due to their inability to defend themselves, their small frame, and lack of autonomy. Fractures are reported to account for 25% of all paediatric injuries [2], including both accidental and suspected physical abuse. Effectively understanding these fracture mechanisms requires an understanding of how developing bone responds to forces and their mechanical properties. However, there are very few experimental studies on the mechanical properties of developing bone; often focused on a specific age range. Forman et al. [3] included seven children aged 1.3–4 years old in their study which utilised three-point-bending experiment at high loading rate, and found that fracture moment increased rapidly during development, noting geometric changes as the major factor. The fracture moments reported in that study were in the range of 61.4–84.8 Nm. Ouyang et al. [4] evaluated the maximum bending force of the femur in ten children from 2–12 years old, conducting a quasi-static three-point-bending experiment on fresh bone free of soft tissues. The reported maximum bending force for the femur was in the range of 15.7–72.6 Nm.

Since it is difficult to obtain whole bone samples from children for mechanical testing, there is a desire to understand the strength and fracture tolerance of developing bone through alternative and non-invasive methods such as image-based finite element analysis (FEA) [5].

Computed tomography to strength (CT2S) is an FEA pipeline developed at the University of Sheffield, originally for the prediction of bone strength (or failure load) in the elderly, before being extended to children [6,7]. CT2S has been used previously to determine the strength in paediatric femurs using quantitative computed tomography (QCT)-based FEA models in a cohort of 35 young children (aged 0–3 years old) [8,9]. However, the process of conducting studies based on the CT2S pipeline is typically time-consuming and requires a well-trained engineer due to the barrier to entry and difficulty presented by computer-aided engineering (CAE) tools. This can present limitations to the application of the pipeline, especially amongst users with a clinical background looking to assess bone strength.

Within the CT2S pipeline, the landmarking of paediatric femurs is a crucial step in setting up a local coordinate system for FEA that provides a consistent region of interest (ROI). Epiphyseal locations are typically used as a reference for landmarking long bones in adults. However, in very young children such as infants, epiphyses are often not yet ossified and therefore not visible with ionising radiation imaging such as computed tomography (CT). Automated methods have previously been developed to address the challenges of landmarking femurs through statistical shape analysis [10–12]. While these methods presented good results, they focused on bones where the epiphyseal centres were visible and did not include any young children under the age of 4-years. Additionally, these models require larger cohort to allow for training, which is limited in younger paediatric data. Operators must therefore rely on training and surrounding features to make a judgement on the epiphyseal locations for paediatric femurs where the epiphyseal centres are not visible. This uncertainty is likely to introduce variability across children of different ages, especially with inexperienced operators.

Therefore, the primary aim of this study is to develop and evaluate an automated architecture for the CT2S pipeline (from meshing to result generation) applied to children. The specific objectives are: (a) to report predicted femoral critical moments on an extended cohort of 69 children and their correlation to age; (b) to create a repeatable and reliable method for automatically identifying the epiphyseal ossification centres; and (c) to quantify the impact of automation on the CT2S pipeline.

## Materials and methods

This study utilised anonymised QCT scans from a cohort of 69 post-mortem children collected at the Sheffield Children’s Hospital, of which 60 were aged 0–3 years in group 1 (G1) and nine were aged 3–15 years in group 2 (G2). The anonymised post-mortem scans were accessed on 2023-10-09. Ethics approval was granted by the West Midlands Edgbaston Research Ethics Committee (REC Reference Number: 15/WM/0242; IRAS project ID: 181203). There was no observable pathology reported that would affect the normal development of the bone. The CT scans were obtained using a GE Lightspeed 64-slice CT scanner. The cohort was scanned with two separate protocols, both protocols used a constant peak tube voltage of 100 kVp and two different constant currents of 59 mA and 100 mA. The scan reconstruction filter was set as medium with the convolution kernel set at standard. In-plane pixel size was 0.33–1.00 mm with an average of 0.68 mm, with a slice thickness and spacing of 0.625 mm. A European spine phantom (ESP) was scanned for each protocol to allow for densitometric calibration. Part of this dataset has been reported previously [8,9]. G1 was further separated into three subgroups: G1a included three children (aged 1–2 weeks) with no ossified femoral epiphyses, G1b included 40 children (aged 0–16 weeks) with ossified distal femoral epiphyses only, and G1c included 17 children (aged 0–3 years) with ossification of both proximal and distal femoral epiphyses.

**Fig 1:**
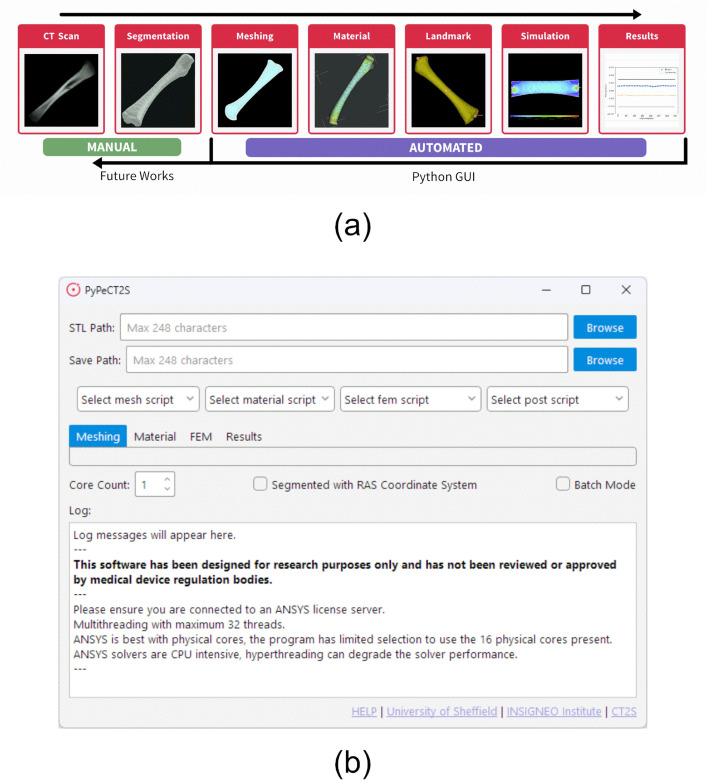
Visualisation and flow of the PyPeCT2S process. **(A)** The PyPeCT2S pipeline and step breakdown. **(B)** GUI of PyPeCT2S [14].

The pythonic paediatric computed tomography to strength (PyPeCT2S) pipeline/toolset was built using *Python* (v 3.11.x), *PyQt6* (v 6.x), *PyMAPDL* (v 0.69.x) [13] in order to present a consistent package to users. Steps within the tool were structured based on their functional roles, enabling definable workflows for end-users (see Fig 1A). The tool dynamically constructed step/tab graphical user interface (GUI) components through a factory function. The layout for the factory function was defined through a dictionary or JSON schema embedded in scripts that were arranged via a folder structure. The simplicity and indirect nature of the GUI allowed for inexperienced users with *Qt* to build new interfaces without code overhauls, large scale time investment, or changes. Additionally, this method supported hot swap functionality in the GUI, where scripts can be inserted or removed without the need to rewrite the program. While the tool was initially built around the ANSYS software suite, it is not limited to ANSYS, and scripts for any CAE suite compatible with *Python* can be developed. In this study, the choice was made to address the processes after segmentation, as automatic segmentation of paediatric cases presents additional challenges around developmental stages and computational requirements for running on an end user machine.

The right femur for each case was segmented using either ITK-SNAP [15] or 3DSlicer [16] to create a solid model of the femur. The segmentations were subsequently processed through the PyPeCT2S pipeline to mesh the bone with quadratic 10-node tetrahedral elements utilising a joint ICEM-CFD and Mechanical APDL 23R2 process (ANSYS INC., PA, USA). Mesh size was determined through convergence analysis depending on the age range. Two representative cases are illustrated here: one at 16-month-old (middle of G1), and one at 8-year-old (middle of G2). The converged mesh size was 2 mm and 3 mm for each case, respectively. The convergence results for the peak first and third principal strains can be seen in S1 and S2 Figs. Material properties were obtained from mapping CT Hounsfield Units to the mesh through Bonemat (Bonemat build 903 ver. 3.2 – 10/12/23) [17], where the elastic modulus was binned in steps of 50 MPa and applied across elements to create a heterogeneous model of the whole bone. The estimation of Young’s modulus (E) was made based on a series of relationships described in the following equations. Eq (1) describes the relationship between greyscale value (grey) and CT density $\left(\rho_{\text{CT}}\right)$ [18,19], where *m* and *c* refer to the slope and intercept determined from the ESP calibration. Eq (2) estimates the ash density from $\rho_{\text{CT}}$ using a derivation from adult bones [18]. Eq (3) estimates *E* from a relationship detailed by Morgan et al. [20].


$$\begin{array}{c} \rho_{\text{CT}}=m\cdot \text{grey}+c \end{array}$$ (1)

$$\begin{array}{c} \rho_{\text{ash}}=0.8772\cdot \rho_{\text{CT}}+0.07895 \end{array}$$ (2)

$$\begin{array}{c} E=14664\rho_{\text{ash}}^{1.49} \end{array}$$ (3)

### Landmark determination

Landmarking is an important precursor to the application of boundary conditions in the simulation of paediatric femurs, which helps to determine a consistent ROI for investigation at different loading orientations. For each paediatric femur, three landmarks were determined: the distal epiphyseal centre ($P_{d}$), the midshaft ($P_{m}$), and the proximal epiphyseal centre ($P_{p}$) based on a previously reported approach [8].

Previously, these three landmarks were manually determined by operators, which could be subjective, and time-consuming. The accuracy is of particular concern in very young children, where the epiphyseal growth plates are not yet fully ossified or fused, as the manual landmarking requires individual operators to visually identify the proximal and distal epiphyseal centres on CT images when they are invisible or only partially visible. In addition, the subjective nature of this approach means that landmark placement is likely to differ between operators, or even the same operator on different occasions, leading to poor reproducibility without extensive training (through personal observation by the authors). The procedure of manual landmarking is also time-consuming, especially when analysing large numbers of images.

**Fig 2:**
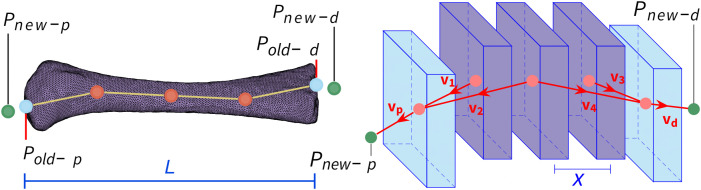
Visual representation of landmark determination. Visual representation of how landmarks are found in the paediatric femur through the automatic landmarking algorithm, estimating the proximal and distal ossification centres of a paediatric femur (age: 2 weeks). $P_{new}$ and $P_{old}$ represent the new and old landmark locations, respectively. Index *p* and *d* refer to the proximal and distal, respectively.

To address these challenges, a new automatic technique was developed, utilising the 3D geometry of the mineralised femur. The proposed new method operates by splitting the femur into a pre-determined number of slices (see Fig 2). Each slice has a centroid, the location of which is determined as the centre of mass of the 2D slice, where each slice is one unit thick (equivalent to one voxel). The presence of an epiphysis at the end of the bone was determined by comparing the mean distance between the centroids $\left(x_{mean}\right)$ against the overall shaft length (*L*) divided by divisions (ξ). When $x_{mean}$ exceeded ξ, it indicated the presence of a proximal/distal epiphyseal centre in the model and no need for adjustment (using Eq (4)). Otherwise, Eq (5) was used to move the outermost centroid position by an adjustment factor (ϕ) along a resultant vector described by the centroids at the two most proximal/distal slices. The adjustment factors (ϕ) for $P_{d}$ and $P_{p}$ were 0.055 and 0.09 respectively; these values were empirically determined through measurements taken from existing CT scans where the ossification centres were visible.


$$\begin{array}{c} {\bar{P}}_{\text{new}}={\bar{P}}_{\text{old}} \end{array}$$ (4)

$$\begin{array}{c} {\bar{P}}_{\text{new}}={\bar{P}}_{\text{old}}+\left(\phi \cdot L\cdot \frac{{\bar{P}}_{\text{n}}-{\bar{P}}_{\text{prev}}}{\left\|{\bar{P}}_{\text{n}}-{\bar{P}}_{\text{prev}}\right\|}\right) \end{array}$$ (5)

Therefore, two landmarking methodologies were investigated in this study: manual and automatic landmarking. For manual landmarking, operators utilised 3D Slicer. If the location of an epiphysis was visible, the centre was placed based on the operator’s best interpretation. If no epiphysis was visible, the centre was approximated based on surrounding visible features and their relative position to the shaft. For automatic landmarking, the landmark positions were determined automatically at runtime of the PyPeCT2S pipeline using Eqs (4) and (5).

To assess the effect of the manual versus automatic landmarking methodologies on predicted bone strength, FEA was conducted on the 60 younger children in G1(including subgroups G1a, G1b, and G1c) using the boundary conditions described in the following section.

### Boundary conditions and finite element results evaluation

**Fig 3:**
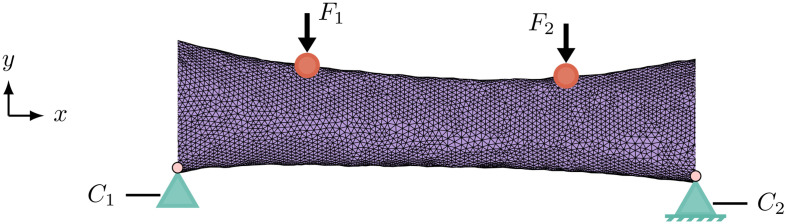
Boundary conditions set up for paediatric bone in a four-point-bending simulation. The section represents 50% of the bone length, as the ROI under investigation.

This study used the four-point bending boundary condition presented in prior works [8,9]. The bone was shortened to the main shaft, by removing the distal and proximal ends. One end of the shaft was fully fixed, while the other end was only partially fixed allowing for translations in the x, y, and z directions. Two equal forces of 120 N ($F_{\text{applied}}=F_{1}=F_{2}$) were applied to the bone in the negative y direction at two locations, equidistant from the centre and end of the bone (see Fig 3).

For each femur, the four-point bending simulation was repeated 35 times by rotating the bone at every 10 ° around the long (x) axis. At each orientation, the first and third principal strains were evaluated within the ROI, which was in between the two points of force application (see Fig 3). The overall critical moment (*M*_critical_) was defined as the moment of the minimum force (*F*_critical_) required to exceed the prescribed elastic strain limits ($\epsilon_{critical}$) of 0.0073 and 0.0104 for tension and compression respectively [21], and being the lowest value across all 36 orientations (see Eqs (6) and (7)). The elastic strain limits used were derived from adult data, which are likely to under-estimate the predicted critical failure values. This was used as a worst-case scenario evaluation in the absence of reliable age-dependent strain limits for children.


$$\begin{array}{c} F_{\text{critical}}=\frac{F_{applied}\cdot \epsilon_{critical}}{\epsilon } \end{array}$$ (6)

$$\begin{array}{c} M_{\text{critical}}=F_{\text{critical}}\cdot \frac{L}{4} \end{array}$$ (7)

### Multi-threading and effective hardware utilisation

Multi-threading code and offloading functionality of *C* based *Python* libraries were used where possible to navigate the challenge of imposed hardware limitations and code base limitations and utilise hardware more effectively. Three methods were utilised during the development of PyPeCT2S to run FEA simulations: A base standard solver, a single simulation pooled method, and multi-simulation batched method.

A hierarchical adaptive methodology that treats certain elements of a single simulation or batch with different priority was used to handle multi-threading. As the task progresses, the tool can close unneeded threads and redistribute them effectively to sub problems within the simulation. This method more effectively utilises available threads at the cost of holding licences. Computational efficiency was further improved by leveraging high speed *Python* and *C* libraries to complete non-finite element (FE) tasks.

To compare PyPeCT2S with the original CT2S pipeline, tests were carried out on timing and similarity of results. A comparison of overall pipeline completion time performed through an experienced and an inexperienced user was used to provide an understanding of overall impact and user-friendliness. Predicted critical moments from the full cohort of 69 children using the PyPeCT2S pipeline were also compared against the previous literature [3,4,9].

## Results

This section first presents the FE predicted principal strain distribution results and then evaluated critical moments with respect to age. In this part, manual landmarks were employed as the benchmark input for FE models, with the rest of the PyPeCT2S pipeline being used to run simulations. Both the younger G1 and the older G2 groups were used, with a total of 69 children included in the analyses.

The second part of the results focuses on the performance of the PyPeCT2S pipeline, by first comparing the manual landmarking with the automatic landmarking method, followed by an evaluation of the efficiency of the new pipeline against the original CT2S. For the manual versus automatic landmarking comparison, G1 (60 cases) was used as they presented more challenges when identifying epiphyseal centres.

### Strain distribution in children at different ages

**Fig 4:**
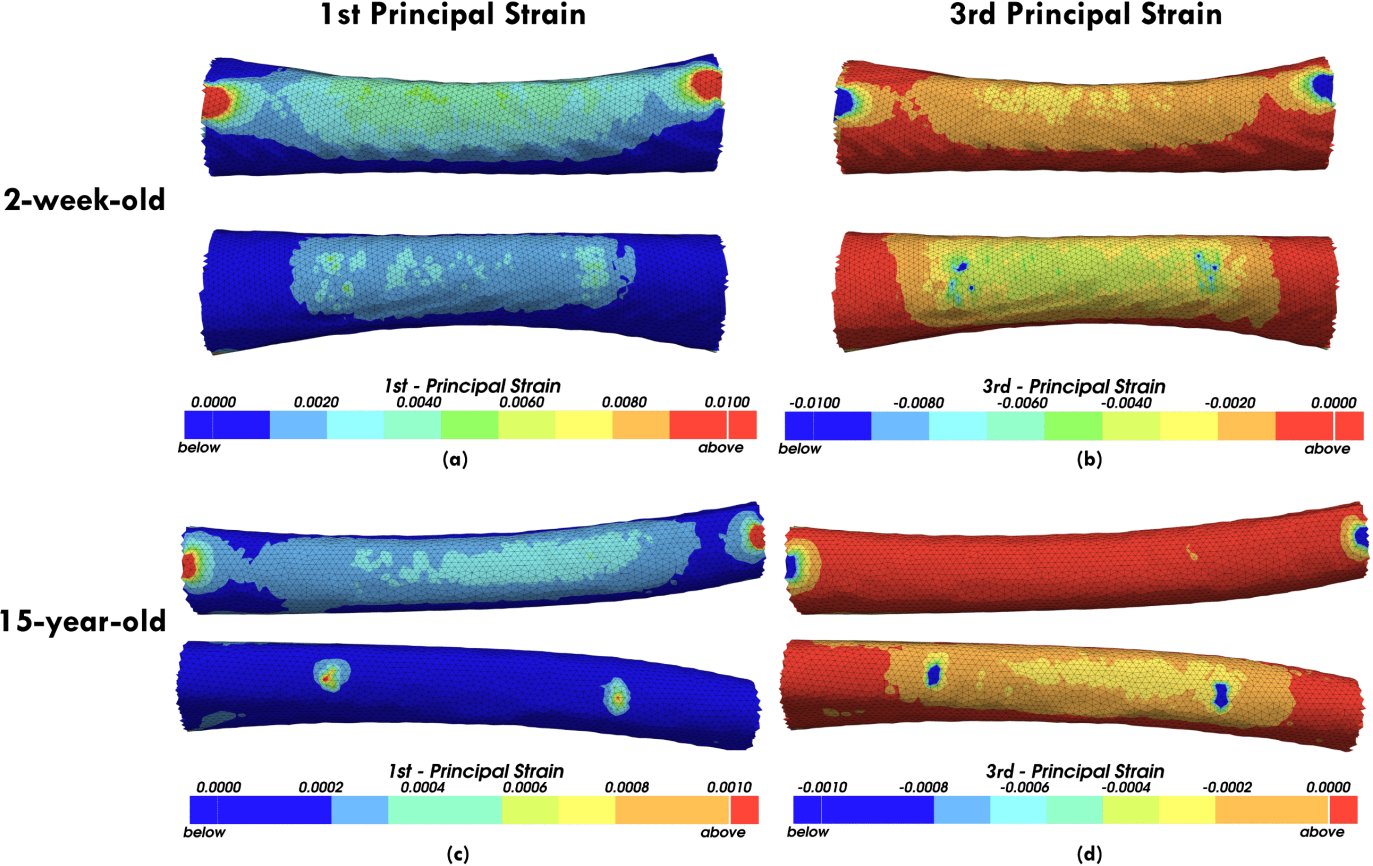
Contour plots of first and third principal strain distributions for two representative cases at different ages. Contour plots of FE predicted first/maximum (left) and third/minimum (right) principal strain distributions for a 2-week-old (a,b) and a 15-year-old (c,d) children showing both the lateral (top) and the medial (bottom) views. Note that the predicted strain values were generally much lower in the 15-year-old, hence the range of strain plotted was also smaller in the older case. Note that the bone models shown are not to scale.

From the FEA results using manual landmarks, strain patterns can be compared between femurs of children at different ages. Two illustrative cases of one 2-week-old and one 15-year-old are shown in Fig 4. Both the first and third principal strain distributions showed similar patterns in these two cases. The predicted strains in the older bone were generally much lower in value compared to the younger child, owing to the higher stiffness in the bone, and consequently a much higher failure load. In addition, the older bone also had comparatively lower first principal strain in the medial view and lower third principal strain in the lateral view, when compared to the younger bone.

### Critical moment versus age

**Fig 5:**
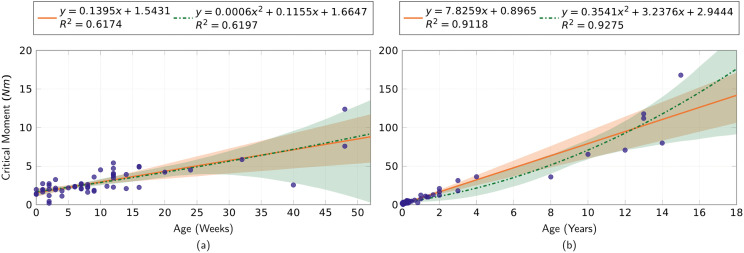
Predicted four-point-bending critical moment (Nm) plotted against age. A: 0 to 1-year-old only (n = 50). B: 0 to 15-year-old (n = 69). The *p* values for all regressions are < 0.001. Both linear and quadratic regressions are banded with a 95% confidence interval.

The FE predicted critical moments generally increased with age, as shown by the regression plots in Fig 5. Regression in the full cohort of 69 (Fig 5B) children had high R^2^ values of 0.91 and 0.93 for the linear and quadratic equations, respectively. The confidence interval became wider towards older ages (e.g., adolescents) due to the smaller sample size in that age group.

**Fig 6:**
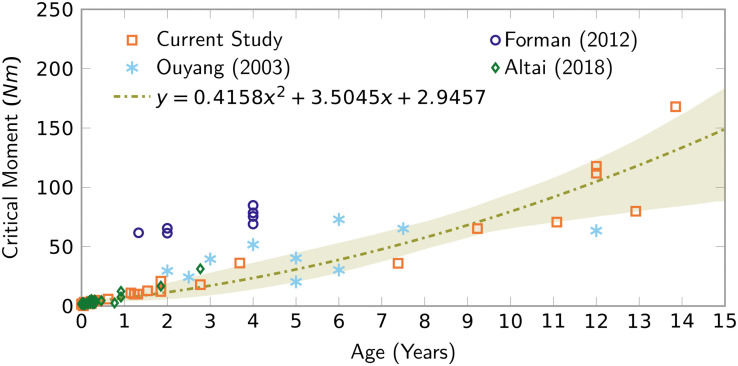
Critical moment for bending against age. Comparing FE predicted critical moment results from the current study to the Altai et al. (2018) [9] study, and those reported from previous experimental studies [3, 4].

The trend of increasing critical moment with age, and the rate of increase, generally agree with previous experimental studies [3,4] (Fig 6).

### Comparison of manual and automatic landmarking

**Fig 7:**
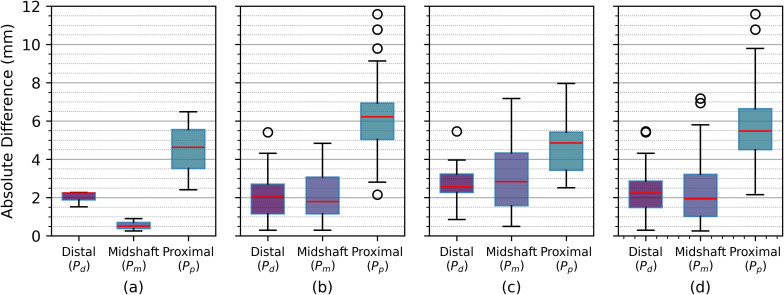
Absolute distance in coordinates between automatic and manual landmarking locations. A: G1a with no visible proximal or distal epiphyseal centre, n = 3. B: G1b with no visible proximal epiphyseal centre but visible distal epiphyseal centre, n = 40. C: G1c with visible proximal and distal epiphyseal centres, n = 17. D: All 60 children under 3-year-old in G1.

The automatic landmarks showed consistent performance over the younger age G1 group (0–3 years) when compared against manual landmarking as the benchmark. The absolute distance in coordinates (manual versus automatic landmarks) for both the distal and midshaft landmarks were comparatively low across all three subgroups (G1a, G1b, and G1c) (see Fig 7). However, the automatically determined proximal landmark had a consistently higher difference compared to the manually determined location. Across all 60 children, the median absolute distance between two landmarking methodologies was 2.24 mm, 1.94 mm, and 5.48 mm for the distal ($P_{d}$), midshaft ($P_{m}$), and proximal ($P_{p}$) epiphyseal centres respectively.

In G1a (Fig 7A), the smallest group with only 3 children, $P_{m}$ showed the strongest agreement between the two landmarking methods with a median difference of 0.52 mm, followed by $P_{d}$, with a median difference of 2.24 mm, whereas $P_{p}$ showed the largest difference (median: 4.63 mm) in this small group. This pattern was consistently seen in G1b and G1c. G1b was the largest amongst the three subgroups, which also contained the largest number of cases with visible $P_{d}$ points (Fig 7B). In G1b, where no proximal epiphyseal centre ($P_{p}$) was present, a large range of absolute distance in $P_{p}$ (2.16–11.59 mm with a median of 6.22 mm) can be seen between automatic and manual landmarks, indicating no clear agreement between the two methods. The differences between $P_{d}$ and $P_{m}$ were comparable in this group, as well as in G1c (Fig 7C), although the spread of $P_{m}$ distance in G1c was larger.

Considering all three subgroups combined (Fig 7D), the wider interquartile range observed at the proximal epiphyseal centre ($P_{p}$) was continually present in all groups, demonstrating the greatest difference between manual and automatic landmarking amongst all three landmark points. Both $P_{m}$ and $P_{d}$ showed small differences between automatic and manual methods, whereas $P_{p}$ showed a consistently larger difference, indicating potential room for improvement for the proximal landmarks.

**Fig 8:**
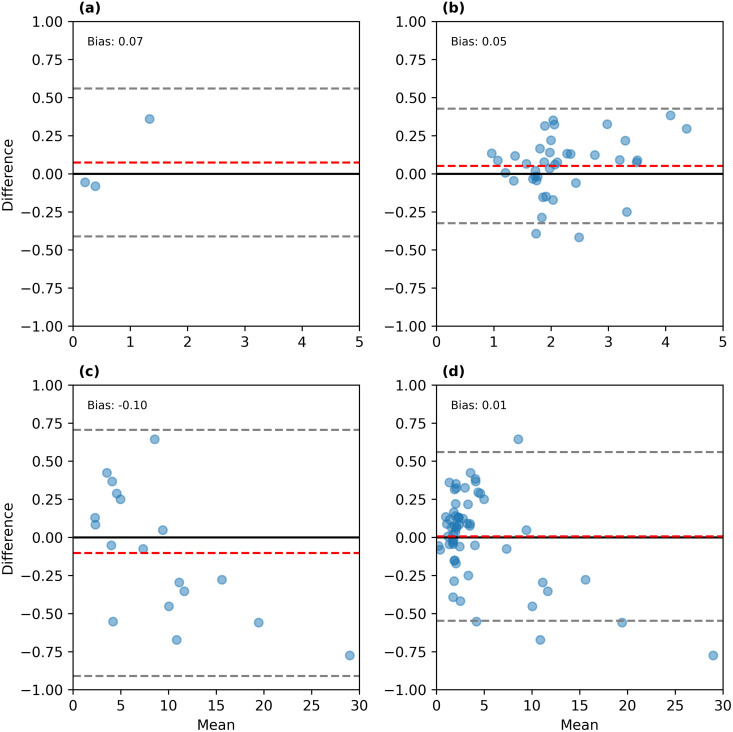
Bland-Altman test results comparing the FE predicted critical moments for the younger G1 group at a representative angle of 110 ° under four-point-bending. A: G1a with no visible proximal or distal epiphyseal centre, n = 3. B: G1b with no visible proximal epiphyseal centre but visible distal epiphyseal centre, n = 40. C: G1c with visible proximal and distal epiphyseal centres, n = 17. D: All 60 cases under 3-year-old in G1.

The Bland-Altman plots for three subgroups are shown in Fig 8, which measure the difference in the predicted critical moment values between manual and automatic landmarking methods against the mean critical moment of both methods. The plots showed small confidence intervals for all 60 children, with minimal bias of 0.07, 0.05, −0.10, and 0.01 for G1a, G1b, G1c, and all children in G1 respectively, suggesting a good level of agreement between the automatic and manual methods.

**Fig 9:**
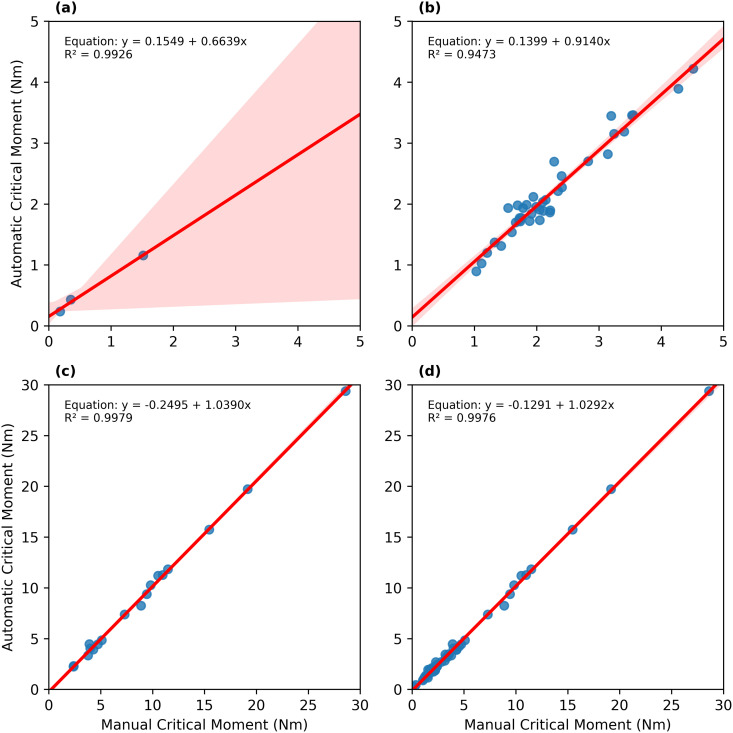
Regression test results for FE predicted critical moments at an angle of 110 ° under four-point-bending. A: G1a with no visible proximal or distal epiphyseal centre, n = 3. B: G1b with no visible proximal epiphyseal centre but visible distal epiphyseal centre, n = 40. C: G1c with visible proximal and distal epiphyseal centres, n = 17. D: All 60 children under 3-year-old in G1.

The regression between the FE predicted critical moments using manual or automatic landmarking methods are shown in Fig 9, at one representative loading orientation under four-point-bending. A high level of agreement was seen in all subgroups, with *R*^2^ values of 0.993, 0.947, 0.998, and 0.998 for G1a, G1b, G1c, and all children in G1 respectively. Note that the fitting in G1a should be cautioned as this subgroup only contained three children. Nevertheless, the results suggested that the differences observed in Fig 7 resulted in minimal differences in FE predictions.

### PyPeCT2S

**Fig 10:**
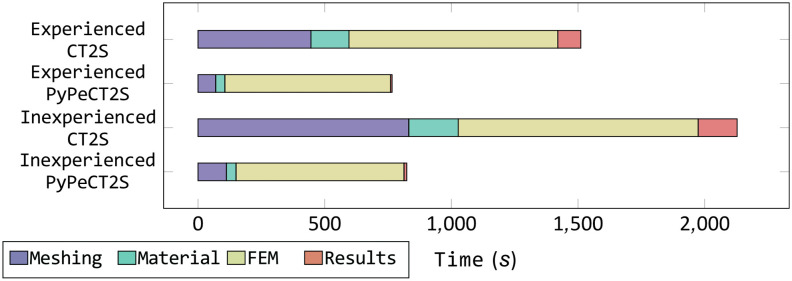
Results for PyPeCT2S and CT2S completion times between one experienced and one inexperienced user. An experienced user was classed as having an engineering degree and receiving guided learning on ANSYS, while an inexperienced user was classed as a non-engineer with no formal training on ANSYS.

Improvements were seen in both the time and ease of using PyPeCT2S against standard ANSYS scripting, even for an experienced operator (see Fig 10). While all steps saw a time reduction using the PyPeCT2S pipeline, the largest improvements were seen in steps surrounding the simulation. The greatest time reduction is seen in the meshing step with an 86.5% and 84.3% reduction between the inexperienced and experienced user, respectively. Overall, the time used to complete the entire pipeline reduced by 49–61% in PyPeCT2S against the original CT2S pipeline, with a time reduction from 25 to 13 min for the experienced user and from 35 to 14 min for the inexperienced user.

## Discussion

An automated and user-friendly pipeline for the creation and simulation of CT-based FE models of paediatric femurs was developed and used to simulate femurs under four-point-bending. The additional issue of landmarking on the paediatric femurs, where epiphyseal centres were not visible, was handled through the creation of an automated algorithm that derived landmarks from a 3D model of the femur. The combination of these two developments allowed for the analysis of FE predicted critical moments in an expanded cohort of children from 0 to 15 years old.

This study presented a sizeable cohort of data in the younger age group of children with a relatively well-spaced age range. However, there is a lack of cases in the older group over 3-year-old, which leads to higher uncertainty in the regression reported, reflected by a large confidence interval. In the previous study by Altai et al. (2018) [9], it was highlighted that regressions suggesting critical moment at birth to be non-zero. Results from the current study confirmed this and indicated low but non-zero bone strength at birth. However, with the current trend of critical moment over age, which was similar to data presented in Forman et al. (2012) [3], it is likely that neither a linear nor quadratic regression is suitable for quantifying the relationship between critical moment and age, and a higher polynomial relationship may be required.

Results in the extended cohort (using benchmark manual landmarks) showed good general agreement (Fig 6) with previous experimental studies conducted on whole cadaveric femurs [3,4]. The current results were slightly lower than those reported in Forman et al. (2012) [3], but agreed well with those reported in Ouyang et al. (2003) [4] across different ages. It is worth noting that there was limited data between 3–18 years of age for all these datasets, which makes it difficult to draw conclusions on this age range. In addition, both Forman et al. (2013) and Ouyang et al. (2003) conducted three-point-bending experiments, while this study used four-point-bending simulations. This means that both experimental studies are likely to result in higher critical moments than the current FE study. The slight under-prediction of our results compared to Forman et al. (2012) in Fig 6 could also be due to the higher loading rate (1.5 ms^-1^) used in Forman’s experiments. This high loading rate is likely to induce some dynamic effects in the experiment, which were not accounted for in the current FE analyses with a quasi-static loading condition. Bone is also a rate-dependent material, which is known to have varying mechanical responses based on the rate at which it is loaded [22]. This dependency is likely to be further amplified by the mixed elasto-plastic nature of paediatric bones. In contrast, Ouyang used a lower loading rate of 0.008 ms^−1^, which can be approximated as quasi-static, leading to a closer match to our FE predictions.

One assumption in the comparison of critical moment especially in the younger age range was the use of adult derived failure limits and density conversion equations in the cohort. Existing experimental studies have shown that paediatric bone is highly ductile and undergoes rapid remodelling during growth [23], although setting a single failure criterion during the entire childhood will be difficult due to changes during development [23]. Paediatric bones are reported to have a higher elastic strain limit. However, there is a range of uncertainties in such reported values owing to the limited number of studies [23,24]. Zimmermann et al. (2019) used a small cohort of one infant and four 2–14 years old children, whereas Ambrose et al. (2018) investigated tibia and rib samples and acknowledged that their strain limits (0.0397 for tension for 0–1 years old) could be overestimated due to the use of three-point-bending. Hence, this study chose to use the more consistently recognised failure limits derived from adult cadaveric femurs, to represent a worst-case scenario estimation. These adult-derived thresholds provide a conservative estimation for failure across the entire cohort. For comparison, the cohort was additionally processed with an elastic strain limit of 0.0397 derived from infant tibiae [24]. As expected, the predicted critical moments increased accordingly based on Eq (6), indicating the bone would potentially sustain a higher load/moment before failure than currently predicted. However, these values resulted in large deviations when compared against previous experimental results on cadaveric femurs (Fig 6), indicating a potential issue in applying infant-based strain limits directly to a wide age range of children. Further investigation in age-dependent, bone-specific paediatric strain limit is therefore needed in the future.

Parallel to this is the assumption to use adult density conversion equations. In the absence of child-specific equations, which remain ethically difficult to obtain, adult cadaveric data remains the viable alternative. This is supported by Öhman et al. (2011) [25], who showed a strong correlation between ash density and Young’s modulus for 4–15 years old, which also continued into adulthood. In addition, ash density was found to be a good predictor of strength for cortical bone in children. This provided confidence that although mechanical properties of bone differed between adults and children, such difference could be quantified through the difference in ash density and reflected through the density to Young’s modulus relationships.

The development of the new automatic landmarking method for paediatric femur presented an acceptable level of accuracy and difference to manual landmarking. While manual landmarks were used as the gold standard baseline, there is no definitive ground truth, especially for infants whose epiphyses are not yet mineralised, as the epiphyseal locations can only be reliably determined using magnetic resonance imaging (MRI) in these cases. As seen in Fig 7, the median absolute distances between manual and automatic landmarks were largely under 2.5 mm, except for the proximal landmark point. In a further analysis, the distances were normalised against the length of each bone, which yielded comparable results. This result is comparable to Xu et al. (2025) [11,12], where a root-mean-square error of approximately 1.78 mm was achieved to predict paediatric femur geometry (4–18 years) using statistical shape-density models, although their study used a different approach by employing principal component analysis and had no younger children under the age of 3 years.

The FEA results showed that despite the differences between automatic and manual landmarks, especially with the larger variation in the proximal location, there was minimal difference in the FE results for critical moment. This is in line with information reported in Eggermont et al. (2024) [10], where despite finding noticeable differences between the determined and original locations on the knee, the resulting strength scores remained accurate and highly correlated to those with the original landmarks. However, Eggermont also used a different method of landmarking, which was based on statistical shape density models that relied on the algorithm to learn population level anatomical variations from a training dataset.

Overall, the automation showed large reductions in processing time in both experienced and inexperienced user groups, showing effective reduction and automation in the pipeline. By bringing both user groups in line with completion times, we saw an approximately 60% reduction (PyPeCT2S versus CT2S) in the time used to run the overall pipeline, bringing completion to 13 min. This is in combination with producing near identical results to those previously reported in our group [8,9].

The current study has several limitations. The number of cases in the older age range (> 3 years) was smaller than the younger age range (0–3 years), leading to a larger spread of data in the regression analyses. We hope to address this in the future by including more cases in the older age range. The study only looked at the performance of one experienced and one inexperienced operator against the algorithm. It will be important in future studies to challenge the algorithm against multiple inexperienced operators, especially those from a clinical background, to establish a more accurate time-saving estimation for the PyPeCT2S pipeline. In addition, future work is also planned to include paired CT and MRI scans to provide a more realistic representation of infants’ femurs by using the MRI scans to obtain non-mineralised epiphysis and register it with the mineralised bone obtained in CT. This would allow for a more reliable identification of the epiphyseal centres, where they have not yet formed or are not yet visible on CT. The additional anatomical fidelity is also essential for expanding the scope of paediatric simulations to include scenarios where boundary conditions or external loads are applied directly to the epiphyseal regions, such as in the studies of classical metaphyseal lesions or slipped capital femoral epiphysis. In these instances, the growth plate acts as a critical mechanical interface due to its substantially lower stiffness compared to mineralised bone. Its presence is likely to alter the load transfer and strain distribution across the metaphysis.

The current study presents a viable method for automating the CT2S pipeline, including the automation of landmarking based on 3D femoral models using simplified vector calculus. The study cohort has been extended to 15 years of age and through automation, the predicted FE results continued to show good correlation with the literature. Due to the inherent challenge in obtaining paediatric bone samples for mechanical testing and evaluation, this work presents important and valuable advancements to investigate bone strength in children through models developed based on non-invasive imaging techniques. It further highlights that the barrier to simulation can be substantially lowered to make it easier to create digital twin models more easily and rapidly, potentially by clinical users. The automated PyPeCT2S pipeline will further enhance our understanding of skeletal development and enrich the scarce data on immature bone mechanics.

## Supporting information

S1 Fig Results for PyPeCT2S convergence analysis on a 16-month-old child. PyPeCT2S mesh convergence analysis was performed using meshes ranging from 1–4 mm in 0.5 mm steps. The converged mesh size is highlighted in green.
(TIF)

S2 Fig Results for PyPeCT2S convergence analysis on an 8-year-old child. PyPeCT2S mesh convergence analysis was performed using meshes ranging from 1–4 mm in 0.5 mm steps. The converged mesh size is highlighted in green.
(TIF)
